# Complex Febrile Seizures As the Initial Presentation of Nutritional Rickets Due to Severe Vitamin D Deficiency

**DOI:** 10.7759/cureus.99346

**Published:** 2025-12-16

**Authors:** Tuqa A Abdulsalam, Mina Almanasir, Gokul Erumbala

**Affiliations:** 1 General Pediatrics, Al Jalila Children's Hospital, Dubai, ARE; 2 Pediatric Emergency Medicine, Al Jalila Children's Hospital, Dubai, ARE

**Keywords:** alkalosis, febrile seizures, hypocalcemia, nutritional rickets, vitamin d deficiency

## Abstract

Simple febrile attacks are common in young children and are typically benign. However, complex febrile seizures are characterized by recurrence, focal features, or longer duration and require assessment to determine underlying causes, such as metabolic issues like hypocalcemia. Vitamin D deficiency rickets, a preventable yet re-emerging disease, can also present with seizures.

We report a previously healthy, exclusively breastfed eight-month-old boy who developed three episodes of general tonic seizures, initially thought to be triggered by an upper respiratory infection. Further workup revealed hypocalcemia (total calcium of 6.8 mg/dL, ionized calcium of 0.91 mmol/L), severe vitamin D deficiency (25-hydroxyvitamin D of 3.5 ng/mL), low phosphate, elevated alkaline phosphatase, and a high serum parathyroid hormone level, all indicative of nutritional rickets. Other factors that may have contributed to the worsening of his ionized calcium included mild respiratory alkalosis. The patient was treated with IV calcium gluconate, followed by maintenance oral calcium and vitamin D. After the correction of hypocalcemia, he had no further seizures and was discharged.

This case underscores the importance of monitoring ionized calcium and vitamin D levels in an exclusively breastfed infant who presents with complex febrile seizures. The sudden decline in ionized calcium, leading to seizures in a susceptible patient, along with fever-induced alkalosis, could be the triggering factors. Prompt diagnosis and appropriate treatment can reduce the risk of neurologic complications from nutritional rickets, thus improving the outcome.

## Introduction

Febrile seizures are the most common form of childhood convulsions, occurring in approximately 2-5% of children between the ages of six months and five years [1]. Although the vast majority of febrile convulsions are benign and classed as simple, convulsions having complex features (i.e., history of focal signs/seizure evolution, recurrence within 24 hours, or presentation lasting longer than 15 min) must be appropriately evaluated for underlying etiologies including infections or structural anomalies of the central nervous system (CNS) infection, or metabolic disturbances (e.g., hypoglycemia, hyponatremia, or hypocalcemia) [1,2].

Hypocalcemia is a known cause of seizures in neonates and infants, which can potentially be underdiagnosed in the context of a febrile seizure. Calcium is an essential component of neuronal excitability and synaptic transmission, and low calcium levels in the blood can reduce the seizure threshold [3]. Nutritional rickets, secondary to vitamin D deficiency, is a known cause of hypocalcemia in infants [4].

Vitamin D deficiency results in a reduction of intestinal calcium absorption, leading to secondary hyperparathyroidism, hypocalcemia, hypophosphatemia, and elevated alkaline phosphatase levels. Prolonged deficiency causes defective bone mineralization and the typical radiographic features of rickets, but earlier symptoms, such as seizures or tetany, can occur without radiographic abnormalities [4,5]. Despite global public health interventions, vitamin D deficiency is now classified as a re-emerging public health issue, particularly among individuals living in areas with low sunlight availability and those with dark skin pigmentation, who often lack supplementation [6].

Maternal and infant vitamin D deficiency rates remain high in the Middle East, as indicated by limited studies, due to cultural clothing practices, limited sun exposure, and reduced intake of vitamin D-fortified foods [6,7]. Both the American Academy of Pediatrics (AAP) and the Pediatric Endocrine Society recommend that all exclusively breastfed infants should receive 400 IU of vitamin D per day starting in the first few days of life to prevent deficiency [4,8].

We present a case of nutritional rickets, who presented to us with complex febrile seizures in an eight-month-old male child, in whom calcium levels were significantly low, along with undetectable vitamin D levels. This case emphasises the importance of metabolic etiologies in atypical presentations of febrile seizure, particularly in individuals with a risk of vitamin D deficiency.

## Case presentation

An eight-month-old boy with a history of febrile seizures since six months of age was brought to the pediatric emergency department (PED) with three generalized tonic seizures during the preceding 24 hours. All the episodes of seizures were accompanied by fever, with the maximum documented fever on presentation being 39°C. The child was a full-term infant who was exclusively breastfed from birth and had reportedly just commenced complementary feeding. He had never been supplemented with vitamin D. He was born via an uncomplicated vaginal delivery with an uneventful perinatal history and never needed NICU care. His developmental milestones were age-appropriate.

The child had symptoms of upper respiratory tract infection (nasal discharge and cough) two days before admission. The first seizure happened on the preceding night of presentation to the PED and lasted between two and three minutes. The mother reported generalized tonic posturing, eye rolling upwards, perioral pallor, and drooling with brief postictal drowsiness. A second episode was observed the next morning at home with the same semiology and duration. A third seizure took place while the child was on the way to the hospital, about thirty minutes before admission.

In the emergency, the child was noted to be conscious and irritable. Upon examination, vital signs were as follows: a temperature of 38.1°C, a heart rate of 186 beats/minute, a respiratory rate of 42 breaths/minute, and oxygen saturation at 98% in room air. The anterior fontanelle was flat and soft, with no focal neurological deficits. Cardio-respiratory and abdominal examination findings were normal. There was no clinical evidence of meningeal irritation. Beading of the costochondral junctions (rachitic rosary) was felt on palpation of the chest wall.

Due to the history of multiple seizures at short intervals, a comprehensive infectious and metabolic workup was initiated. Laboratory studies revealed severe hypocalcemia, with a serum calcium level of 6.8 mg/dL (reference range: 8.6-11 mg/dL) and an ionized calcium level of 0.91 mmol/L (reference range: 1.15-1.29 mmol/L). Low phosphate of 3.9 mg/dL and high alkaline phosphatase (1,113 U/L) were evidenced. The vitamin D status was severely low, 3.5 ng/mL (reference: 30-100 ng/mL), and the parathyroid hormone (PTH) level was high (437 pg/mL), indicating secondary hyperparathyroidism. Serum electrolytes, renal function, and magnesium levels were normal (Table 1).

**Table 1:** Bone profile upon admission

Parameter	Patient value	Reference range
Total calcium (mg/dL)	6.8	8.6 – 11.0
Ionized calcium (mmol/L)	0.91	1.15 – 1.29
Phosphate (mg/dL)	3.9	4.6 – 7.9
Alkaline phosphatase (U/L)	1,113	122 – 469
25-OH vitamin D (ng/mL)	3.5	30 – 100
Parathyroid hormone (pg/mL)	437	8.5 – 61.3
Magnesium (mg/dL)	2.04	1.7 – 2.3

**Figure 1:**
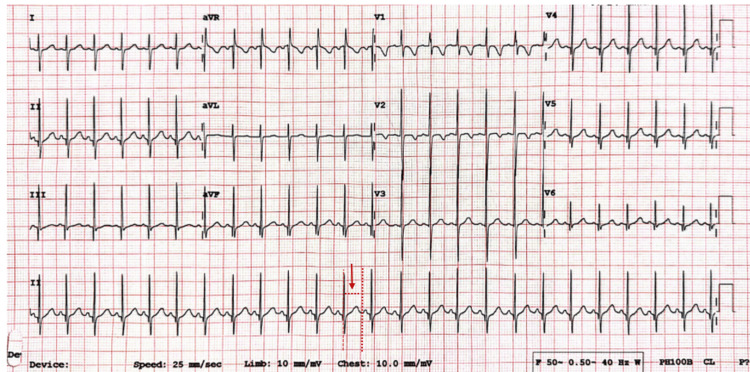
Twelve-lead electrocardiogram demonstrating normal sinus rhythm with a corrected QT interval (QTc) of 405 milliseconds within normal limits (arrow)

The arterial blood gas analysis revealed slight metabolic acidosis (pH=7.359, bicarbonate (HCO_3_-)=19.9 mmol/L). The QT interval was not prolonged on the electrocardiogram (Figure 1).

Cerebrospinal fluid (CSF) was clear and colorless with 1 white cell/μL (normal glucose and protein and negative multiplex meningitis/encephalitis PCR panel). Blood, urine, and CSF cultures were negative. Nasopharyngeal respiratory viral PCRs were negative, and Enteropathogenic E coli was positive on gastrointestinal PCRs.

**Figure 2:**
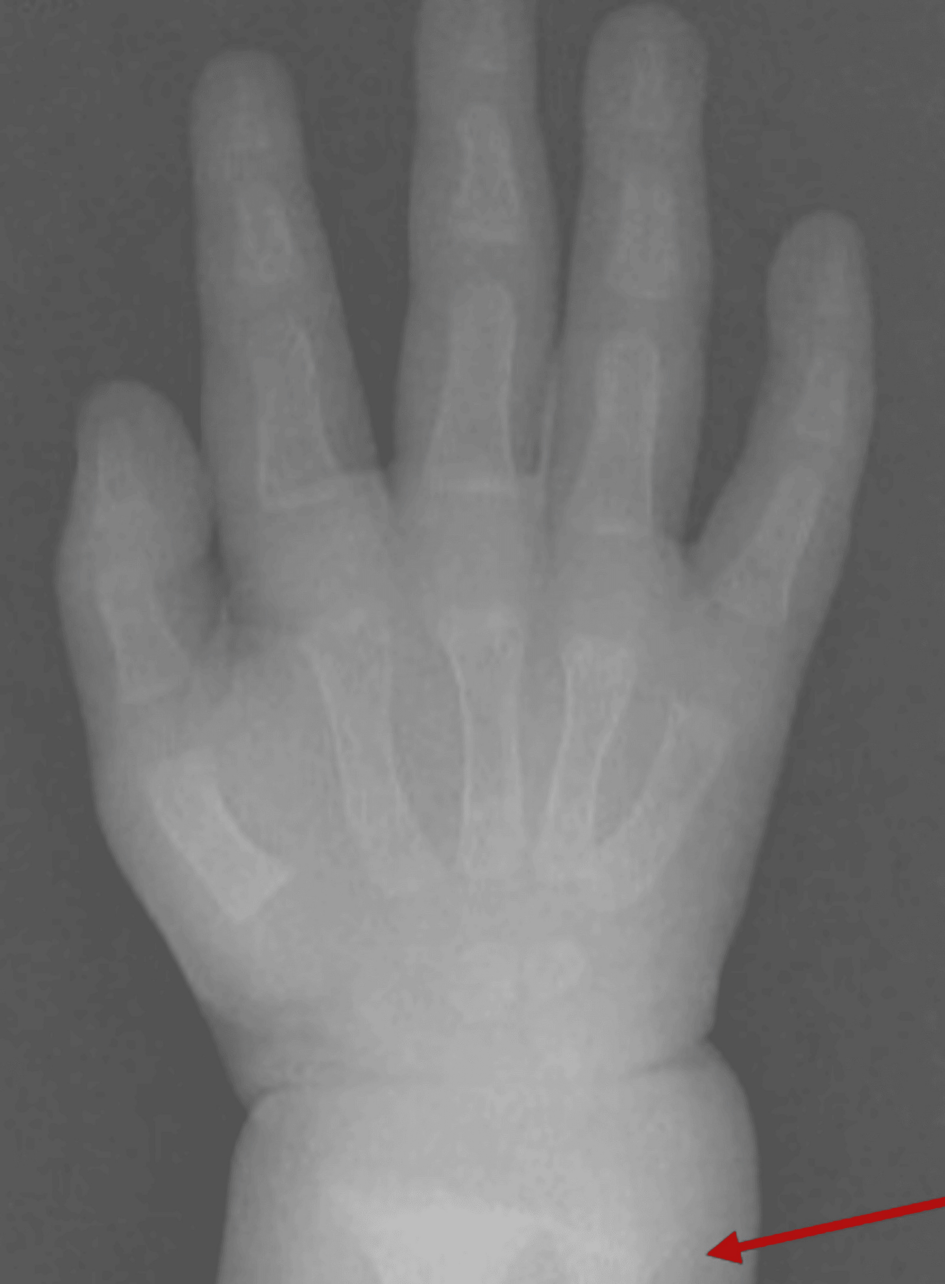
Wrist X-ray showing metaphyseal cupping and fraying (arrow), consistent with active rickets

X-ray of the wrist (Figure 2) showed classic features of active rickets, including cupping, fraying, and widening of the distal metaphyses of the radius and ulna, consistent with vitamin D deficiency-related rickets.

**Figure 3:**
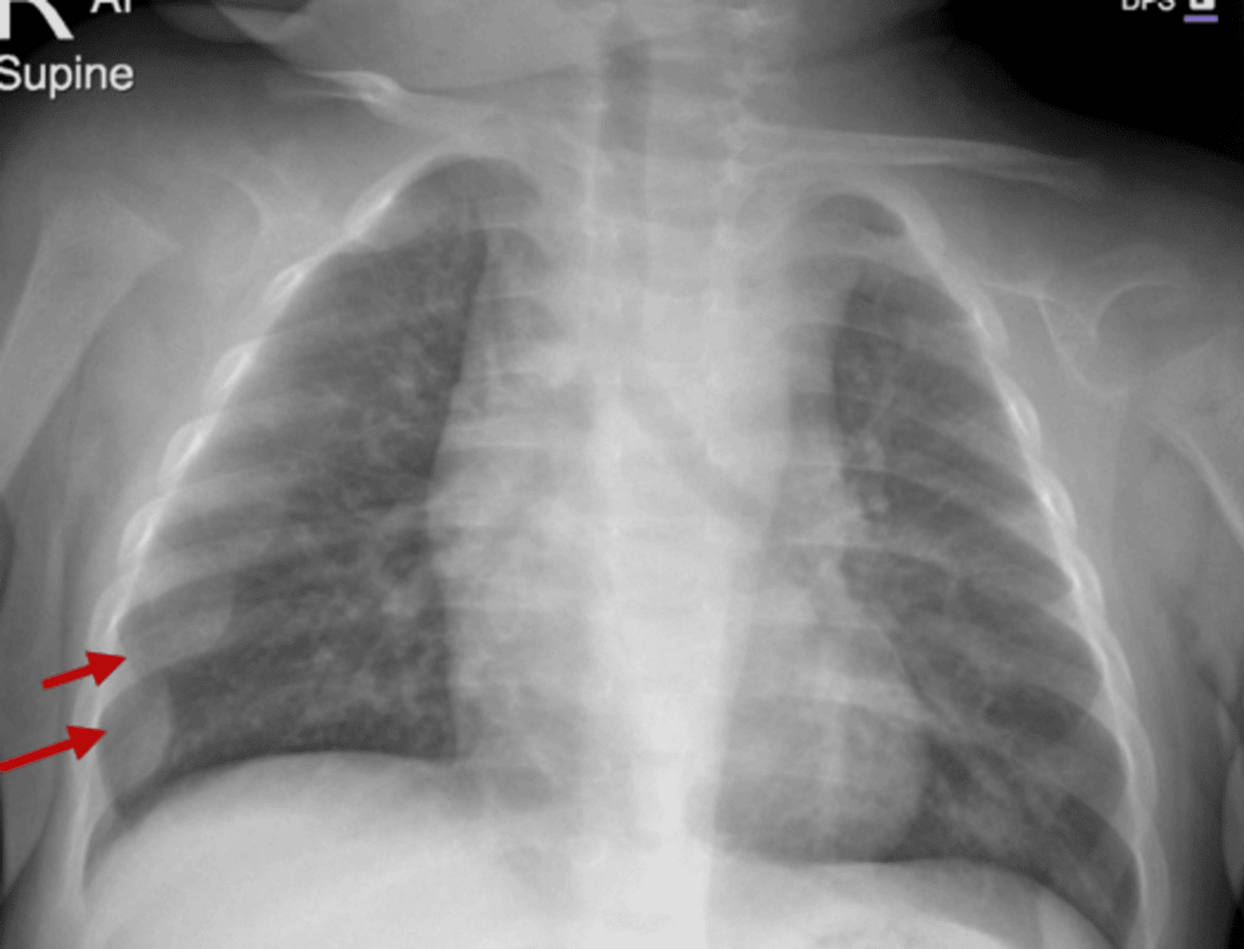
Chest X-ray showing mild rachitic rosary (arrows) and osteopenia

Furthermore, chest X-ray (Figure 3) showed mild prominence of the costochondral junctions (rachitic rosary) and generalized osteopenia. These findings supported the clinical diagnosis of nutritional rickets.

In the acute setting, the patient was treated with IV calcium gluconate 30 mg/kg elemental calcium, which resulted in an improvement in the ionized calcium level of 0.97 mmol/L after six hours. He commenced on oral calcium (elemental calcium 50 mg/kg/day in four divided doses). He received a stat dose of vitamin D3 (colecalciferol) 25,000 IU, followed by a daily maintenance dose of 1,000 IU. Serial calcium monitoring every six hours showed a slow, albeit steady improvement, with ionized calcium increasing to 1.01 mmol/L without further IV administration.

The patient was hemodynamically stable and afebrile throughout his inpatient stay. No further seizures were observed. He was not found to have a focal bacterial infection, and his inflammatory markers were normal. The patient was advised to continue taking oral vitamin D and calcium with a plan to follow up as an outpatient, with repeat imaging planned to assess for signs of rickets radiographically. The mother was educated on the importance of maternal vitamin D testing and supplementation, as the child was exclusively breastfed.

The patient was discharged from the hospital on the fourth day of hospitalization in good general condition. The discharge medication was daily colecalciferol 1,000 IU and elemental calcium 50 mg/kg/day. The final diagnosis was complex febrile seizures secondary to hypocalcemia due to severe vitamin D deficiency, consistent with nutritional rickets.

## Discussion

Febrile seizures (FS) constitute the most common form of neurologic disorder in young children and occur in 2% to 5% of children aged six months to five years. Most febrile seizures are benign and classed as simple febrile seizures, which are often self-limited. However, complex febrile seizures, defined by focal signs, long duration (>15 minutes), or recurrence within 24 hours, need to be further investigated to rule out CNS infections, structural diseases, or metabolic disorders such as hypoglycemia, hyponatremia, and hypocalcemia [1,2].

Hypocalcemia is a well-known yet often overlooked trigger of seizures in the young pediatric population. Calcium plays a critical role in neuronal excitability, the stabilization of the resting membrane potential, and synaptic transmission. A decrease in serum calcium levels might raise the seizure threshold by increasing neuronal membrane permeability [3,4]. In febrile patients, this risk is increased, as fever can also contribute to neuronal hyperexcitability. Thus, in children with complex FS or afebrile seizure of obscure infectious source, serum calcium must be determined in the initial evaluation.

An additional and often overlooked contributor is respiratory alkalosis, which can occur during febrile illness due to hyperventilation. Alkalosis enhances the binding of albumin to calcium, resulting in a low concentration of ionized calcium even when the total calcium concentration is normal [5]. The venous blood gas in this patient showed a pH of 7.359 and a CO₂ tension (pCO₂) of 34.8 mmHg, confirming the evidence of mild respiratory alkalosis. These metabolic derangements, coupled with an already existing deficiency in vitamin D and a borderline ionized calcium (0.91 mmol/L), probably prompted the seizure event. This description illustrates the fact that even mild hypocalcemia can unmask itself clinically, under alterations in the metabolic status [5,6].

Although preventable, nutritional rickets secondary to severe vitamin D deficiency remains a significant cause of hypocalcemia in infants worldwide. Vitamin D increases intestinal calcium and phosphate absorption; deficiency results in defective mineralisation and secondary hyperparathyroidism and abnormalities in biochemistry, including low calcium, low phosphate, and high alkaline phosphatase [5,6]. In this patient, the laboratory studies were compatible with the classical biochemical profile of calcipenic rickets (hypocalcemia, hypophosphatemia, highly elevated serum alkaline phosphatase, and a very low 25-hydroxyvitamin D level [3.5 ng/mL]). Moreover, the highly increased parathyroid hormone (437 pg/mL) would reflect a compensatory secondary hyperparathyroidic response [7].

The child's exclusive breastfeeding and lack of vitamin D supplementation put him at a high risk for deficiency. Given that human breast milk is low in vitamin D, such a diet would not provide an adequate amount of the vitamin, especially in settings with limited sun exposure, dark skin pigmentation, or maternal nutritional deficiency [8]. The AAP, Endocrine Society, and Global Consensus Guidelines on Nutritional Rickets all recommend universal supplementation of 400 IU/day of vitamin D to all breastfed infants beginning in the first few days of life [9-11]. Yet, today, compliance with these recommendations is poor, and discrepancies between practice and recommendations in many areas still hamper routine postnatal supplementation.

Notably, the decrease of ionized calcium (0.91 mmol/L) in this patient was relatively small but was sufficient, along with the onset of fever and hyperventilation, to trigger the seizures. This highlights the importance of clinical awareness of neurological symptoms in patients, even with mild to moderate hypocalcemia, during periods of metabolic and infective stressors.

The radiologic manifestations of rickets are frequently delayed compared with the biochemical abnormalities, and findings can be subtle or absent in early disease. However, mild rachitic rosary was clinically evident in this case, and radiographs of the wrist/chest were planned to assess the splaying of metaphyses characteristic of rickets [12]. Early diagnosis and correction of early biochemical abnormalities constitute the mainstay of therapy. The timely identification and correction of the biochemical abnormalities in this child through a standardised management protocol [6,13] resulted in the prompt resolution of symptoms in this child.

From a public health perspective, this case highlights the value of preventive measures such as prenatal maternal vitamin D testing, supplementation for breastfed infants, and caregiver education. For infants with complex or atypical febrile seizures, the index of suspicion for nutritional deficiencies should be kept high, particularly in areas with a relatively high prevalence of rickets.

## Conclusions

This case emphasizes the need to consider metabolic causes, especially vitamin D deficiency hypocalcemia, in infants with atypical febrile seizures. Despite being preventable, nutritional rickets continues to be an ongoing cause for hypocalcemic seizures in exclusively breastfed children not supplemented with vitamin D, particularly in settings with limited sunlight exposure and cultural practices that adversely impact vitamin D metabolism.

Early diagnosis and rapid correction of calcium and vitamin D levels are crucial in avoiding the risk of recurrence or long-term neurological sequelae. This case contributes to the growing evidence supporting the practice of routine vitamin D supplementation in infants, public health promotion, and raising awareness of this emerging health concern.

## References

[1] Berg AT, Shinnar S (1996). Complex febrile seizures. Epilepsia.

[2] Basatemur E, Sutcliffe A (2015). Incidence of hypocalcemic seizures due to vitamin D deficiency in children in the United Kingdom and Ireland. J Clin Endocrinol Metab.

[3] Han P, Trinidad BJ, Shi J (2015). Hypocalcemia-induced seizure: demystifying the calcium paradox. ASN Neuro.

[4] Cooper MS, Gittoes NJ (2008). Diagnosis and management of hypocalcaemia. BMJ.

[5] Lee JW (2010). Fluid and electrolyte disturbances in critically ill patients. Electrolyte Blood Press.

[6] Munns CF, Shaw N, Kiely M (2016). Global Consensus Recommendations on prevention and management of nutritional rickets. J Clin Endocrinol Metab.

[7] Misra M, Pacaud D, Petryk A, Collett-Solberg PF, Kappy M (2008). Vitamin D deficiency in children and its management: review of current knowledge and recommendations. Pediatrics.

[8] Holick MF (2007). Vitamin D deficiency. N Engl J Med.

[9] Dawodu A, Akinbi H (2013). Vitamin D nutrition in pregnancy: current opinion. Int J Womens Health.

[10] Wagner CL, Greer FR (2008). Prevention of rickets and vitamin D deficiency in infants, children, and adolescents. Pediatrics.

[11] Institute of Medicine (2011). Dietary Reference Intakes for Calcium and Vitamin D. National Academies Press.

[12] Pettifor JM (2004). Nutritional rickets: deficiency of vitamin D, calcium, or both?. Am J Clin Nutr.

[13] Kirkgoz T, Guran T (2018). Primary adrenal insufficiency in children: diagnosis and management. Best Pract Res Clin Endocrinol Metab.

